# Assessment of Implicit Gender Bias During Evaluation of Procedural Competency Among Emergency Medicine Residents

**DOI:** 10.1001/jamanetworkopen.2021.47351

**Published:** 2022-02-07

**Authors:** Ashley See, Michael Pallaci, Adam R. Aluisio, Jenny Beck-Esmay, Michael Menchine, Michael Weinstock, Chun Nok Lam, Jeff Riddell

**Affiliations:** 1 Department of Emergency Medicine, Adena Health Systems, Chillicothe, Ohio; 2 Department of Emergency Medicine, Kettering Health, Dayton, Ohio; 3 Department of Emergency Medicine, Adena Health System, Chillicothe, Ohio; 4 Department of Emergency Medicine, Summa Health System, Akron, Ohio; 5 Department of Emergency Medicine, Alpert Medical School, Brown University, Providence, Rhode Island; 6 Department of Emergency Medicine, Mt Sinai Morningside, New York, New York; 7 Department of Emergency Medicine, Keck School of Medicine, University of Southern California, Los Angeles; 8 Department of Emergency Medicine, Adena Health Systems, Chillicothe, Ohio; 9 Department of Emergency Medicine, The Wexner Medical Center, Ohio State University, Columbus; 10 Department of Population and Public Health Science, Keck School of Medicine, University of Southern California, Los Angeles

## Abstract

**Importance:**

Gender disparities exist throughout medicine. Recent studies have highlighted an attainment gap between male and female residents in performance evaluations on Accreditation Council for Graduate Medical Education (ACGME) milestones. Because of difficulties in blinding evaluators to gender, it remains unclear whether these observed disparities are because of implicit bias or other causes.

**Objective:**

To estimate the magnitude of implicit gender bias in assessments of procedural competency in emergency medicine residents and whether the gender of the evaluator is associated with identified implicit gender bias.

**Design, Setting, and Participants:**

A cross-sectional study was performed from 2018 to 2020 in which emergency medicine residency faculty assessed procedural competency by evaluating videos of residents performing 3 procedures in a simulated environment. They were blinded to the intent of the study. Proceduralists were filmed performing each procedure from 2 different viewpoints simultaneously by 2 different cameras. One was a gender-blinded (ie, hands-only) view, and the other a wide-angled gender-evident (ie, whole-body) view. The faculty evaluators viewed videos in a random order and assessed procedural competency on a global rating scale with extensive validity evidence for the evaluation of video-recorded procedural performance.

**Main Outcomes and Measures:**

The primary outcome was to determine if there was a difference in the evaluation of procedural competency based on gender. The secondary outcome was to determine if there was a difference in the evaluations based on the gender of the evaluator.

**Results:**

Fifty-one faculty evaluators enrolled from 19 states, with 22 male participants (43.1%), 29 female participants (56.9%), and a mean (SD) age of 37 (6.4) years. Each evaluator assessed all 60 procedures: 30 gender-blinded (hands-only view) videos and 30 identical gender-evident (wide angle) videos. There were no statistically significant differences in the study evaluators’ scores of the proceduralists based on their gender, and the gender of the evaluator was not associated with the difference in mean scores.

**Conclusions and Relevance:**

In this study, we did not identify a difference in the evaluation of procedural competency based upon the gender of the resident proceduralist or the gender of the faculty evaluator.

## Key Points

**Question:**

Does gender disparity exist in evaluation of procedural competency for emergency medicine (EM) residents?

**Findings:**

This cross-sectional study enrolled 51 EM faculty from 19 states who evaluated 60 videos of 10 EM residents performing 3 procedures. Each procedure was shot with a gender-blinded view and a gender-evident view, and there was no significant difference in the EM faculty evaluations of procedural competency based on gender.

**Meaning:**

These findings suggest that there was no difference in the evaluation of procedural competency based on the gender of the resident proceduralist or the gender of the faculty evaluator.

## Teaser

This cross-sectional study evaluates implicit gender bias in assessments of procedural competency in emergency medicine residents and assesses whether the gender of the evaluator is associated with gender bias.

## Introduction

Gender disparities exist throughout the field of medicine, including in resident milestone assessments,^1,2^ academic rank,^3,4^ authorship in medical journals,^5^ invited commentaries,^6^ and salary.^7,8^ While the number of women entering medical school now exceeds that of men,^9^ women remain significantly underrepresented as faculty in academic medicine, particularly at advanced academic ranks.^3,10^ Female academic physicians report experiencing significant gender bias in their careers.^11^

Residency training may be a pivotal time when gender bias influences career decisions.^12^ In addition to qualitative differences in the kind of feedback that emergency medicine (EM) residents received from attending physicians, recent studies have highlighted an attainment gap between male and female EM residents in evaluations of performance on the Accreditation Council for Graduate Medical Education (ACGME) milestones.^2,13^ Dayal et al^2^ found that milestone ratings were higher for men than women at graduation while similar at entry into residency. A large study^1^ examining longitudinal ACGME milestone ratings for all EM residents over 4 years found men were rated as performing better than women at graduation for 3 procedural subcompetencies, including their general approach to procedures. Proposed explanations include implicit gender bias of faculty evaluators and actual gender-based differences in performance or skill. These gender-based differences may be attributable to the cumulative effects of disadvantages, such as reduced opportunities to access longitudinal mentorship, obtain meaningful feedback, or observe gender concordant role models.^2,14^

Disambiguating between these potential causes of observed disparities is vital to developing interventions to improve gender equity. Blinding evaluators to the gender of the proceduralist would seemingly eliminate implicit bias; this has been tried successfully in other academic domains,^15,16^ but has been pragmatically difficult to perform in the assessment of clinical tasks.

In this study, we estimate the magnitude of implicit gender bias in the assessment of procedural competency in EM residents using a novel, video-based strategy with the gender of the resident hidden vs apparent. Further, we assess whether the gender of the evaluator is associated with any identified implicit gender bias.

## Methods

**Figure 1.**
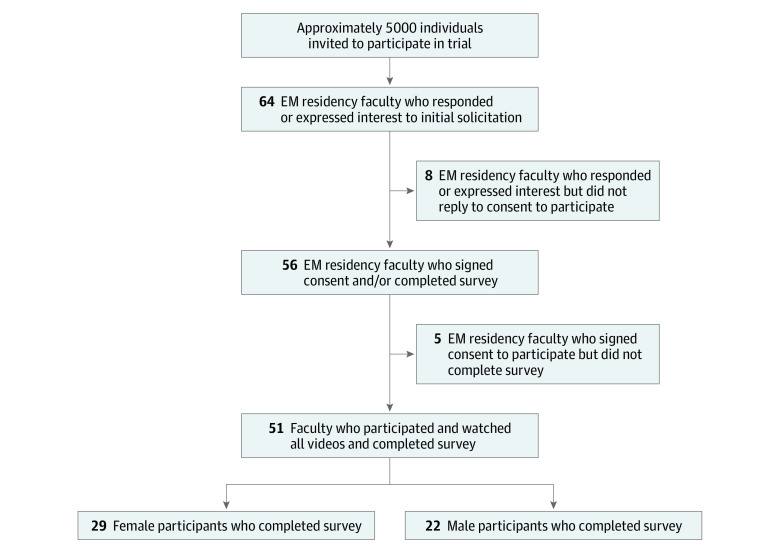
Flow Diagram of Study Participation

We performed a cross-sectional study with EM faculty evaluators to assess implicit gender bias in procedural evaluations of EM residents between 2018 to 2020. This study was approved by the institutional review board of the Adena Hospital System. Informed consent was obtained from each evaluator before receiving the procedure video link. This study followed the Strengthening the Reporting of Observational Studies in Epidemiology (STROBE) reporting guideline. Figure 1 contains the study flow diagram.

### Development of Study Materials

Residents from a single EM residency program served as proceduralists. The sample was chosen for parity among men and women and distribution across training years. Gender was determined by the proceduralists’ self-identified designation. The proceduralists consisted of 5 men (1 postgraduate year [PGY] 1, 2 PGY2s, and 2 PGY4s) and 5 women (2 PGY1s, 1 PGY2, 1 PGY3, and 1 PGY4). After providing informed consent, a standard script was read to the proceduralists, informing them that they would be performing a simulated lumbar puncture, thoracostomy tube, and internal jugular central venous catheter under ultrasonography guidance (eAppendix 1 and eAppendix 2 in the Supplement). They were blinded to the intent of the study.

### Creation of Video Media

**Figure 2.**
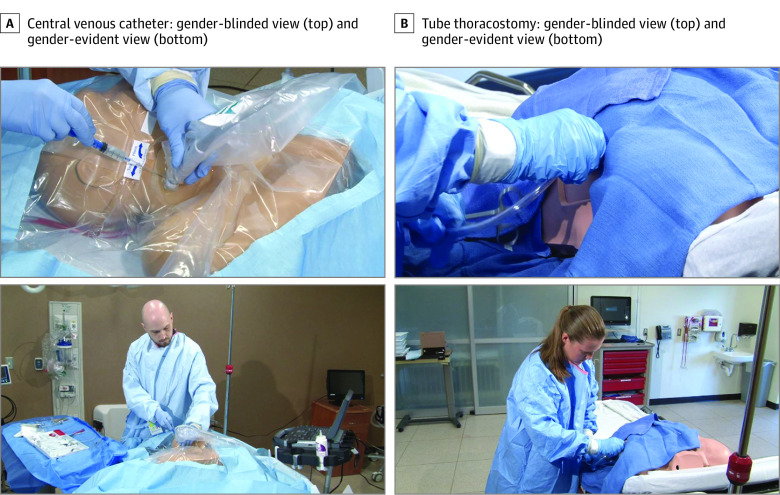
Gender-Blinded and Gender-Evident Views of Emergency Medicine Procedures

For each procedure, 2 high-resolution video cameras simultaneously captured 2 different views of each procedure, including a hands-only view, which concealed the gender of the proceduralist (ie, gender-blinded view), and a whole-body view that included the face, thorax, arms, and hands of the proceduralist, as well as the mannequin (ie, gender-evident view) (Figure 2). Proceduralists removed all jewelry and donned surgical latex gloves and surgical gowns with elastic wrist cuffs and a second pair of opaque blue gloves over the latex gloves, covering the wrist cuffs to reduce the chance of revealing gender in the gender-blinded view. A professional videographer filmed the procedures. A previous study^17^ validated the effectiveness of this method in blinding evaluators to the gender of proceduralists.

Three procedures were performed by each of the 10 proceduralists (ie, 30 procedures); each procedure had a gender-blinded view and a gender-evident view for a total of 60 videos. Each video was assigned a unique number and entered into a log. The raw video was then edited using editing software to remove sound and any nonprocedure-related footage.

### Recruitment of Evaluators

Attending physicians with experience supervising residents and evaluating procedural competency were recruited as study participants through an invitation on the Council of Residency Directors in Emergency Medicine (CORD) ListServ and emails sent by the authors to professional academic contacts in EM. The participants were told that our purpose was to “study evaluation of resident competency in simulated procedures,” but were blinded to the intent of the study. They were provided approximately $100 per hour for their time. Review and scoring of all 60 of the videos took approximately 10 hours.

### Data Collection

Each video media file was numbered, and a randomized sequence of videos was created. After providing consent, each EM faculty evaluator (study participant) was sent a link to a survey generated from SurveyMonkey and unique to the evaluator (eAppendix 3 in the Supplement). The participants were required to enter their demographic information once, watch each of the 60 videos in their entirety (attestation required for each video), and answer the evaluation questions for the procedure. Each evaluator viewed the videos in the same order. They had the option to review the videos in a single session or multiple shorter sessions.

### Procedure Assessment Scoring: The Global Rating Scale

Evaluators scored procedures on a modified global rating scale (GRS) adapted from the Objective Structural Assessment of Technical Skills (OSATS), an assessment with extensive validity evidence for evaluating video-recorded procedural performance^18,19^ in procedures including tube thoracostomy, lumbar puncture, and central venous access. For each procedure video, the participants were required to select a score on a Likert-type scale of 1 to 5 that best reflected their evaluation of procedural performance for each of 6 domains: (1) respect for tissue: (2) time and motion; (3) knowledge of equipment; (4) instrument handling; (5) flow of procedure; and (6) knowledge of the procedure. The use of assistants domain was omitted. GRS in simulation-based assessments is more dependable and may better capture nuanced elements than checklist-based assessments, so we chose the GRS portion of the OSATS.^20,21,22^ Scores on the 6 domains were summed and then divided by 6 to generate the overall score.

### Statistical Analysis

The primary outcome was the difference in mean scores obtained from the gender-blinded view and the gender-evident view between male and female proceduralists. The underlying hypothesis was that implicit gender bias would be associated with a greater difference in scores between the gender-blinded and gender-evident scores for female proceduralists than their male counterparts.

For the secondary outcome, we evaluated differences in blinded vs unblinded scores across proceduralist gender according to the gender of the evaluators. This analysis generated 4 unique comparative groups: female evaluator and female proceduralist, female evaluator and male proceduralist, male evaluator and female proceduralist, and male evaluator and male proceduralist. Scores were summarized using mean evaluation ratings based on grouping derived from the gender of the evaluator and proceduralist (eg, mean of female evaluator for female proceduralist). The resulting scores were not independent because the individual proceduralists performed multiple procedures and the evaluator assessed multiple procedures performed by a limited number of proceduralists. To account for this, we modeled the difference between gender-evident and gender-blinded (dependent variable) pairs of videos with a mixed-effects model that included fixed effects for each evaluator and random effects for each proceduralist using the xtmxed command in STATA. Data were analyzed using Stata 16 (Statacorp).

The key independent variable was proceduralist gender. A significant coefficient for this variable indicates that scores are significantly different between the genders when the gender is revealed as opposed to concealed. We likewise modeled the difference between gender-evident vs gender-blinded using an interaction term between reviewer and proceduralist gender to determine if any potential reviewer gender bias exists or was associated with the gender of the reviewer.

Existing studies have shown a change of 10% per year in GRS scores as residents progress through training.^23^ As such, we estimated that a 10% difference in GRS scores is clinically meaningful. We calculated an a priori study size to detect an absolute difference of at least 10% between the assessment scores for the blinded and unblinded videos. A baseline score of 60% (3 on the Likert scale) was used with a 15% variance based on prior research using the OSATS GRS.^23^ Using α = .0125 (based on a Bonferroni correction for multiple testing) and a power of 90%, the projected sample size required was 21 male reviewers and 21 female reviewers assuming independence of the observations by reviewers. Although independence is likely violated in this study design, the assumption yields conservative estimates for the needed sample size. Statistical analyses were performed by study investigators who were blinded to the gender of the evaluators and the gender of the proceduralist. Statistical analysis was performed in November 2021, significance was set at α = .05, and tests were 2-sided for the multilevel model.

## Results

Fifty-one EM faculty (mean [SD] age, 37 [6.4] years; 29 [56.9%] women; 22 [43.1%] men) evaluated 60 videos each. Geographically, the highest number came from the midwest and northeast regions, where many EM residency programs are concentrated. Thirty-eight of 51 evaluators (74.5%) work as core faculty, while 13 of 51 (25.5%) are clinical faculty. (Table 1)

**Table 1.** Video Evaluator Characteristics

Characteristic	No. (%)	
	Women	Men
Total (N = 51)	29 (56.9)	22 (43.1)
Age, mean (SD)	37.1 (6.3)	38.5 (6.6)
Years in EM practice, mean (SD)	7 (6)	7.8 (5.6)
Years of faculty experience, mean (SD)	6.4 (6.2)	6.5 (4.4)
Role		
Program director	2 (6.9)	2 (9)
Associate program director	11 (38)	3 (13.6)
Core faculty	9 (31)	11 (50)
Clinical faculty	7(24.1)	6 (27.4)
Teaching setting		
Clinical	5 (17.2)	2 (9)
Didactics and clinical	24 (82.8)	20 (91)

Abbreviation: EM, emergency medicine.

Regarding the primary outcome, in the mixed-effects regression model, the male proceduralist gender was not associated with a greater score difference than the female proceduralist gender (*B* = 0.05 [95% CI,−0.20 to 0.29]). The mean (SD) score for women in the gender-evident view was 3.65 (0.52), compared with 3.53 (0.67) in the gender-blinded view (difference, 0.12 [95% CI, −0.04 to 0.29]). For men, the mean (SD) score was 3.75 (0.48) in the gender-evident view, compared with 3.69 (0.51) in the gender-blinded view (difference, 0.06 [95% CI, −0.06 to 0.19]) (Table 2).

**Table 2.** Difference in Mean Scores Between Female and Male Proceduralists Across All Evaluators

Proceduralists	Score, mean (SD)		Difference (95% CI)
	Gender evident (n = 9180)^a^^,^^b^	Gender blind (n = 9180)^a^^,^^c^	
Women	3.65 (0.52)	3.53 (0.67)	0.12 (−0.04 to 0.29)
Men	3.75 (0.48)	3.69 (0.51)	0.06 (–.06 to 0.19)
Women vs men	NA	NA	0.06 (–0.10 to 0.13)

Abbreviaton: NA, not applicable.

^a^ n = 9180 indicates the total number of evalutations.

^b^ Whole-body view.

^c^ Hands-only view.

Similarly, the gender of the evaluator was not associated with the difference in mean (95% CI) scores. There was no significant interaction between the gender of the evaluator and the gender of the proceduralist (*B* = −0.04 [95% CI, −0.22 to 0.14]). Male evaluators scored female proceduralists slightly lower when the gender was evident vs blinded (mean [95% CI], 3.74 [3.60 to 3.89] vs 3.56 [3.38 to 3.73]; difference, 0.19 [95% CI, −0.03 to 0.41]). This was similar to the difference observed for male evaluators scoring male proceduralists (3.77 [95% CI, 3.61 to 3.93] vs. 3.68 [95% CI, 3.52 to 3.84]; difference, 0.09 [95% CI, −0.12 to 0.31]) (Table 3).

**Table 3.** Mean Scores (by Individual Domain) of Female and Male Proceduralists by Female and Male Evaluators (Secondary Outcome)

Evaluator gender groups	Score, mean (95% CI)		Difference (95% CI)
	Gender evident (n = 9180)^a^^,^^b^	Gender blind (n = 9180)^a^^,^^c^	
Female evaluators			
Female proceduralists	3.64 (3.41 to 3.87)	3.53 (3.23 to 3.82)	−0.11 (−0.48 to 0.25)
Male proceduralists	3.72 (3.47 to 3.97)	3.72 (3.44 to 4.00)	0.00 (−0.37 to 0.37)
Female vs male proceduralists	NA	NA	0.11 (−0.04 to 0.23)
Male evaluators			
Female proceduralists	3.74 (3.60 to 3.89)	3.56 (3.38 to 3.73)	0.19 (−0.03 to 0.41)
Male proceduralists	3.77 (3.61 to 3.93)	3.68 (3.52 to 3.84)	0.09 (−0.12 to 0.31)
Female vs male proceduralists	NA	NA	0.10 (−0.02 to 0.20)

Abbreviation: NA, not applicable.

^a^ n = 9180 indicates the total number of evalutations.

^b^ Whole-body view.

^c^ Hands-only view.

## Discussion

We conducted a cross-sectional study to assess implicit gender bias in the evaluation of EM resident procedural competency, using a validated approach to blinding evaluators to the gender of the proceduralists.^17^ Each of the 51 faculty evaluators assessed 60 procedure videos (30 gender-blinded and 30 gender-evident), recording evaluations of 6 unique domains on a Likert scale for a total of 18 360 data points. Based on the perceived and documented prevalence of gender bias in medicine in general^13^ and EM specifically,^2^ we hypothesized that there would be a greater difference in scores for female proceduralists. However, we found no significant difference.

This finding might not be surprising given the conclusions of existing studies. In a systematic review, Klein et al^14^ found that only 5 of 9 included studies demonstrated gender bias, though none were prospective. In a retrospective single-center study of EM residents performing simulated procedures, there was no difference in evaluations associated with resident gender.^24^ Other studies showed slower milestone progression for female EM residents during residency^2^ and small differences in 6 of 22 ACGME milestone subcompetencies.^1^

The discrepancies may be related to differences in assessment data. The 2 large studies^1,2^ that showed gender disparities in procedural evaluations were based on ACGME milestone data from end-of-semester aggregate milestone ratings. These assessments are determined every 6 months by a clinical competency committee (CCC) composed of several faculty members who synthesize multiple sources of assessment information (eg, end of shift evaluations, procedure logs, in-training examination scores, direct observation checklist, and other workplace-based narrative assessments) into a score on each of the 23 milestone subcompetencies.^25^ These summative assessments often rely on incomplete, retrospective information and are often made in an ad hoc manner based on physicians’ gut feelings of performance.

Our study, along with another study of simulated procedures,^24^ used data from direct observations of procedural performance using a checklist or rating form, not on aggregate milestone data; neither found evidence of implicit gender bias. This discrepancy suggests that the bias observed in large milestone data may enter the assessment process downstream from the direct observation of procedural performance. Alternatively, the CCCs making the aggregate assessments in the 2 large milestone studies may not have had access to robust direct observation data.

Most real-world faculty evaluations are inherently less objective than checklist-based procedural evaluations. However, existing tools for direct observations of EM milestones lack validity and reliability. While some argue that subjective expert judgments by medical professionals are unavoidable and should be embraced as the core of assessment of medical trainees,^26^ the complexity of how gender bias manifests in subjective resident assessments remains a significant hurdle.^14^ Further, procedural competencies are a small subset of the 23 EM milestones. While objectifying procedural assessments seems like low-hanging fruit, implicit bias may be more insidious and difficult to gauge in the review of competencies with less reliable evaluation methods. The fact that we found no implicit bias in procedural assessments in our study does not suggest it does not exist in other aspects of residency training.

Our findings might also be explained, in part, by progress in reducing the impact of implicit bias among EM faculty. There have been many recent efforts to decrease the impact of implicit gender bias in medicine, including educational programs addressing implicit bias that have been shown to change faculty attitudes toward women in academic medicine. EM has been a more progressive specialty, with committees from the American College of Emergency Physicians, American Association of Women Emergency Physicians, the Society of Academic Emergency Medicine’s Academy for Diversity and Inclusion in Emergency Medicine and Academy for Women in Academic Emergency Medicine, and the American Academy of Emergency Medicine Women in Emergency Medicine Section working to promote both awareness and change regarding gender issues. Importantly, Females Working in Emergency Medicine has a web-based Women in Medicine educational curriculum and hosts a yearly conference dedicated to providing resources and support for women in EM. While our study might provide some hope, there is still considerable work to be done. While we do not suggest neglecting implicit bias training, it may be more fruitful to focus research and resources on developing interventions aimed at the other systemic and institutional level contributors to gender disparities.

### Limitations

This study had limitations. Our faculty cohort may have different levels of implicit bias than other EM faculty; our faculty evaluators were 56% women, which is higher than the 28% female faculty across academic EM. While this could bias our study, our results are consistent with other studies that found no significant differences based on the gender of the evaluator.^2^ While our study participants were mostly core faculty who sit on CCCs, they were a relatively junior cohort with a mean age of approximately 38 years. While some might hypothesize that a cohort of more senior faculty would have different levels of implicit bias, previous studies show only small correlations between age and implicit or explicit gender bias.

The proceduralists were somewhat unbalanced in terms of their level of experience, with the men being slightly more experienced than the women. This reflects the balance of our residency program, which had more men in the later PGYs and more women in the earlier PGYs. However, the difference in scores between men and women was not significant.

We did not account for viewer fatigue when assigning the videos. There were almost 10 hours of videos, which may have resulted in decreased vigilance toward the end of the session. Additionally, the videos were viewed in the same order by all the evaluators, which introduces the possibility of viewer fatigue blunting the difference on the same videos for each evaluator.

The gender-blinded and gender-evident videos were shot at the same time from different angles. The evaluators may have noticed familiar movements or errors that made them recognize that they were evaluating the same procedure twice. The Hawthorne effect could have played a role in creating a type II error that fails to demonstrate implicit bias that truly existed. However, the evaluators were blinded to the purpose of the study, and the sheer number of procedures to review combined with the random order likely prevented most, if not all, evaluators from drawing this conclusion.

Regarding our assessment tool, there is minimal consensus on what represents a significant difference in scores on the OSATS GRS. Multiple studies have used the GRS, but they are heterogeneous, use multiple variations of the GRS (sometimes in combination with other measures including checklists), and use different GRS targets. We chose a measure that was intuitive based on an extensive review of previous research using the GRS. As such, our sample size may be less than ideal. Further, to parallel what happens in real-world settings, we chose to do minimal faculty training on GRS use. Faculty may have misinterpreted or misapplied the tool, though previous studies have shown that evaluator training did not significantly improve reliability.

## Conclusions

We found no differences in the assessment of procedural competency based on the gender of the proceduralist or the gender of the faculty evaluators. These findings suggest that implicit gender bias in the direct observation of simulated procedures is unlikely to be the source of established gender disparities.

## Notes

Supplement. **eAppendix 1.** Video Consent Form **eAppendix 2.** Script Read to Proceduralists Prior to Performance of Recorded Simulated Procedure **eAppendix 3.** Survey Completed by Study Participants
