# Supplementary material for: Assessment of Implicit Gender Bias During Evaluation of Procedural Competency Among Emergency Medicine Residents
Source: JAMA Netw Open. 2022 Feb 7;5(2):e2147351. doi: 10.1001/jamanetworkopen.2021.47351 (PMC8822382; doi:10.1001/jamanetworkopen.2021.47351)
Supplement: Supplement. — eAppendix 1. Video Consent Form eAppendix 2. Script Read to Proceduralists Prior to Performance of Recorded Simulated Procedure eAppendix 3. Survey Completed by Study Participants [file jamanetwopen-e2147351-s001.pdf]

Supplemental Online Content

See A, Pallaci M, Aluisio AR, et al. Assessment of implicit gender bias during evaluation of procedural competency among emergency medicine residents. *JAMA Netw Open*. 2022;5(2):e2147351. doi:10.1001/jamanetworkopen.2021.47351

eAppendix 1. Video Consent Form

eAppendix 2. Script Read to Proceduralists Prior to Performance of Recorded Simulated Procedure

eAppendix 3. Survey Completed by Study Participants

This supplemental material has been provided by the authors to give readers additional information about their work.

© 2022 See A et al. *JAMA Network Open.*

Appendix 1: Video Consent Form

Without expectation of compensation or other remuneration, now or in the future, I hereby give my consent to Blue Skies HD and ARMC medical education, its affiliates and agents, to use my image and likeness in its publications and research.

This consent includes, but is not limited to: (Initial where applicable)

_________ - (a) Permission to film, photograph, tape, or otherwise make a video reproduction of me;
_________ - (b) Permission to distribute copies from the film(s) (or excerpts of such films), photograph(s), tape(s) or reproduction(s) of me, in part or in whole, for the purpose of research.

This consent is given in perpetuity, and does not require prior approval by me.

Name: ____

Signature:____

Address: ____

Date: ______________________

The below signed parent or legal guardian of the above-named minor child hereby consents to and gives permission to the above on behalf of such minor child.

Signature: ________________________________ Print Name: ______________________________

The following is required if the consent form has to be read to the parent/legal guardian:
I certify that I have read this consent form in full to the parent/legal guardian whose signature appears above.

________________ ____
Date **Signature** of Organizational Representative or Community Leader

© 2022 See A et al. *JAMA Network Open.*

eAppendix 2: Script Read to Proceduralists Prior to Performance of Recorded Simulated Procedure

You are going to be performing a (chest tube/central line/lumbar puncture). No feedback will be provided until after the procedure is completed. You will not be evaluated based on your performance.

While this is a learning experience for you, the session will be recorded for research purposes. Your name will not be used at any time, and the videos will be deleted when the research study is completed.

Informed consent has been obtained from the simulated patient, and the patient has been prepped and draped in a sterile fashion.

Begin the procedure at the point of skin anesthesia. The procedure is over when

- Chest tube: you've secured the tube and hooked it up to the pleurevac
- Central line: you've secured the line and the ports have been drawn and flushed
- Lumbar puncture: you've removed the needle and applied the bandage

Please remove any jewelry from your hands. You will be wearing a surgical gown, and a pair of surgical gloves with a pair of non-surgical gloves over the top.

Do you have any questions?

© 2022 See A et al. *JAMA Network Open.*

eAppendix 3: Survey Completed by Study Participants

Procedural Competency in Simulated Environment

1. Information about you

1. Please enter your first and last initial followed by the first number of your address (ex. TT7)

2. Indicate the abbreviation for the state in which you practice (ex: OH)

3. Please indicate your gender
- Female
- Male

4. Age

5. Years in EM Practice (excluding residency)

6. Years of experience as EM Faculty

7. Please Indicate if you are...
- Program Director
- Associate Program Director
- Core Faculty
- Clinical Faculty

8. Which of the following best describes your teaching setting
- Didactics
- Clinical
- Didactics and Clinical
- None of the above

1

9. With what level of trainees do you typically work?
- Medical Students
- Graduate Medical Education
- MD/DO residents
- Other (please specify)
- Non-physician providers
- All of the above

Procedural Competency in Simulated Environment

2. Instructions

Thank you for your participation in this study.

You will be reviewing 60 videos of resident performed procedures in a simulation lab. The resident training level ranges from PGY1-PGY4.

You will score each video using a Likert-type scale ranging from 1-5. Prompts for a score of 1, 3 and 5 are provided for you. If you feel that a score is between two numbers, such as 2 or 4, you may select the rating that best reflects your evaluation. You will be using the Global Rating Scale for your evaluation. The Global Rating Score consists of 6 domains: Respect for tissue, Time and Motion, Knowledge of Equipment, Instrument Handling, Flow of Procedure and Knowledge of Procedure.

It is very important that you watch the entire video before scoring. You may watch and score the video's all at once or in sessions. Your email address is linked to the survey and it will take you back to where you left off.

Prior to beginning the procedure, the residents were informed that: Consent has been obtained from the patient. The resident was instructed to begin the procedure from the point of skin anesthesia.

Once you have completed your review of each video, please do not go back and change your answers.

Procedural Competency in Simulated Environment

3.

2

© 2022 See A et al. *JAMA Network Open.*

*

1.

☐ I have reviewed the entire video

* 2. Respect for tissue

1	2	3	4	5
Frequently used unnecessary force on tissue or caused damage by inappropriate use of instruments		Careful handling of tissue by occasionally caused inadvertent damage		Consistently handled tissue appropriate with minimal damage
○	○	○	○	○

* 3. Time and Motion

1	2	3	4	5
Many unnecessary moves		Efficient time/motion but some unnecessary moves		Clear economy of movement and maximum efficiency
○	○	○	○	○

3

* 4. Knowledge of Equipment

1	2	3	4	5
Frequently selects wrong instrument or used inappropriate equipment		Selected correct instrument or equipment most of the time		Obviously familiar with the instruments and equipment
○	○	○	○	○

* 5. Instrument handling

1	2	3	4	5
Repeatedly makes tentative or awkward moves with instruments by inappropriate use of instruments		Competent use of instruments but occasionally appeared stiff or awkward		Fluid moves with instruments and no awkwardness
○	○	○	○	○

* 6. Flow of Procedure

1	2	3	4	5
Frequently stopped procedure and seemed unsure of next move		Demonstrated some forward planning with reasonable progression of procedure		Obviously planned course of operation with effortless flow from one move to the next
○	○	○	○	○

* 7. Knowledge of Procedure

1	2	3	4	5
Missed critical steps in the procedure		Completed all important steps of the procedure		Demonstrated familiarity with all aspects of the procedure
○	○	○	○	○

© 2022 See A et al. *JAMA Network Open.*
